# Genomic evidence of recent European introgression into North American farmed and wild Atlantic salmon

**DOI:** 10.1111/eva.13454

**Published:** 2022-08-24

**Authors:** Ian R. Bradbury, Sarah Jean Lehnert, Tony Kess, Mallory Van Wyngaarden, Steven Duffy, Amber M. Messmer, Brendan Wringe, Silje Karoliussen, J. Brian Dempson, Ian A. Fleming, Monica Favnebe Solberg, Kevin A. Glover, Paul Bentzen

**Affiliations:** ^1^ Fisheries and Oceans Canada Northwest Atlantic Fisheries Centre St. John's NL Canada; ^2^ Fisheries and Oceans Canada Bedford Institute of Oceanography Dartmouth NS Canada; ^3^ Centre for Integrative Genetics Norwegian University of Life Sciences Ås Norway; ^4^ Department of Ocean Sciences, Ocean Sciences Centre Memorial University of Newfoundland St John's NL Canada; ^5^ Population Genetics Research Group Institute of Marine Research Bergen Norway; ^6^ Department of Biological Sciences University of Bergen Bergen Norway; ^7^ Biology Department Dalhousie University Halifax NS Canada

**Keywords:** aquaculture, Atlantic salmon, European ancestry, genetic, hybridization, management

## Abstract

Gene flow between wild and domestic populations has been repeatedly demonstrated across a diverse range of taxa. Ultimately, the genetic impacts of gene flow from domestic into wild populations depend both on the degree of domestication and the original source of the domesticated population. Atlantic salmon, *Salmo salar*, used in North American aquaculture are ostensibly of North American origin. However, evidence of European introgression into North American aquaculture salmon has accumulated in recent decades, even though the use of diploid European salmon has never been approved in Canada. The full extent of such introgression as well as the potential impacts on wild salmon in the Northwest Atlantic remains uncertain. Here, we extend previous work comparing North American and European wild salmon (*n* = 5799) using a 220 K SNP array to quantify levels of recent European introgression into samples of domestic salmon, aquaculture escapees, and wild salmon collected throughout Atlantic Canada. Analysis of North American farmed salmon (*n* = 403) and escapees (*n* = 289) displayed significantly elevated levels of European ancestry by comparison with wild individuals (*p* < 0.001). Of North American farmed salmon sampled between 2011 and 2018, ~17% had more than 10% European ancestry and several individuals exceeded 40% European ancestry. Samples of escaped farmed salmon similarly displayed elevated levels of European ancestry, with two individuals classified as 100% European. Analysis of juvenile salmon collected in rivers proximate to aquaculture locations also revealed evidence of elevated European ancestry and larger admixture tract in comparison to individuals collected at distance from aquaculture. Overall, our results demonstrate that even though diploid European salmon have never been approved for use in Canada, individuals of full and partial European ancestry have been in use over the last decade, and that some of these individuals have escaped and hybridized in the wild.

## 1 INTRODUCTION

Escape events from Atlantic salmon, *Salmo salar*, net‐pen aquaculture occur frequently (Diserud et al., 2019; Glover et al., 2019; Keyser et al., 2018), and evidence from across the North Atlantic has repeatedly demonstrated interbreeding and subsequent introgression between wild Atlantic salmon and escaped domestic individuals (Glover et al., 2017; Karlsson et al., 2016; McGinnity et al., 2003; Wringe et al., 2018). The conclusion that introgression between wild and escaped domestic individuals genetically alters wild salmon and reduces population viability has been supported through both experimental and modeling studies in North America and Europe (Bradbury et al., 2020; Castellani et al., 2018; Fleming et al., 2000; McGinnity et al., 2003). Accordingly, escaped farmed salmon and subsequent genetic interactions have been identified as a major threat to the persistence and stability of wild Atlantic salmon across the North Atlantic (Forseth et al., 2017).

Ultimately, the impact of escapees on wild population fitness likely depends on the degree of maladaptation of domestic populations to a given wild environment (Baskett et al., 2013; Fleming, 1995), which can be influenced by both the magnitude of domestication selection and the source population used for domestication. Atlantic salmon domestication has resulted in significant phenotypic (Bolstad et al., 2021; Glover et al., 2018; Skaala et al., 2019) and allelic (Karlsson et al., 2011; Liu et al., 2017; Wringe et al., 2019) differences from wild populations. However, the degree of domestication varies across the Atlantic; in Europe, Atlantic salmon have been subjected to domestication selection for approximately twice as many generations as in North America (Glover et al., 2017). In addition to differences in domestication, studies over the last few decades have consistently revealed substantial genome‐wide genetic differentiation between European and North American salmon (Bourret et al., 2013; Jeffery et al., 2017; King et al., 2001). Recent genomic analysis has revealed these trans‐Atlantic differences are associated with genomic regions ranging in size from single loci (SNPs) to large genomic regions (1–3 Mbp) on four chromosomes (Lehnert et al., 2020). Moreover, these genomic regions exhibit population genomic patterns consistent with natural selection, and have been associated with metabolic, developmental, immune, and neural processes potentially representing adaptive trans‐Atlantic differences and supporting suggestions for subspecies designation in Atlantic salmon (Lehnert et al., 2019, 2020). Collectively, the evidence for greater domestication and significant trans‐Atlantic adaptative differences in salmon from North America and Europe support the hypothesis that the degree of maladaptation could be elevated for European escaped farmed salmon in North America compared with North American farmed salmon (DFO, 2013).

Despite the potential for significant maladaptation of European farmed salmon in the wild in North America, there has been repeated interest in utilizing Norwegian‐origin Atlantic salmon in both Canada and the United States (Baum, 1998; DFO, 2013, 2016, 2018a). European salmon were first imported into Maine from the 1980s until it was prohibited in the 1990s (Baum, 1998). Since then, evidence continues to suggest that the presence of European ancestry in Canadian farmed Atlantic salmon has remained even though diploid European salmon have never been approved for use in Canada (O'Reilly et al., 2006; Porter et al., 1998). Liu et al. (2017) reported that ~27% of Canadian domestic salmon examined in their study had more than 5% European ancestry. O'Reilly et al. (2006) reported the presence of European microsatellite alleles in aquaculture escapees collected at two locations in the Bay of Fundy. Following on this work, DFO (2018b) reported that European farm escapees, or their offspring, appear to have been spawning in the Bay of Fundy during most years between 1997 and 2012. However, to date, few studies have examined the prevalence of European ancestry among North American aquaculture salmon and most detections have been based on small panels of microsatellite loci, with few diagnostic alleles that are likely to significantly underestimate European contributions.

Our goal here is to use recently available genomic resources in conjunction with large collections of wild and domestic Atlantic salmon, to evaluate the hypothesis that European alleles continue to be present in North American domestic salmon and that escapees with European ancestry have been interbreeding with wild salmon in Atlantic Canada. Specifically, we (1) build on recent genomic comparisons between North American and European Atlantic salmon (i.e., Lehnert et al., 2020) to screen North American aquaculture samples collected between 2011 and 2018 for European ancestry; (2) similarly, screen escaped farmed salmon and wild salmon collected throughout Atlantic Canada for evidence of European ancestry to document the extent of impacts of European introgression on wild salmon throughout the region; and (3) finally, explore the genomic distribution of European ancestry across the genomes of introgressed individuals comparing hybrids collected near aquaculture cage locations with introgressed individuals from a naturally occurring region of historical trans‐Atlantic secondary contact. Admittedly, our primary European genomic baseline, which is a large dataset of wild Norwegian Atlantic salmon, does not fully capture diversity present in Norwegian farmed salmon or more broadly in European salmon. As such, we compare the results to a sample of known Norwegian farmed salmon and an additional genomic dataset encompassing the three large European phylogenetic groups (Bourret et al., 2013). This work extends previous studies identifying post‐glacial secondary contact between European and North American salmon (Bradbury et al., 2015; Lehnert et al., 2019) and demonstrates how both the magnitude and the distribution of divergence across genomes can be used to distinguish introgression due to post‐glacial colonization and recent aquaculture escapees.

## 2 METHODS

### 2.1 Sampling

**FIGURE 1:**
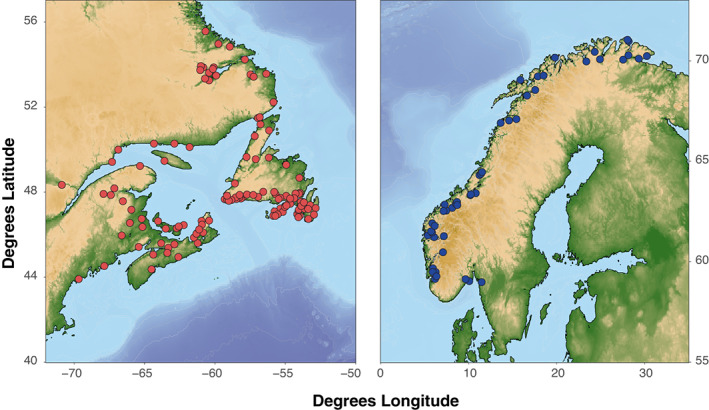
Map of baseline Atlantic salmon sample locations from North America (red, 142 sites, *n* = 4993) and Europe (blue, 50 sites, *n* = 806).

Genotype data for wild salmon were combined from a previously published source (i.e., Lehnert et al., 2020) and newly collected samples for a total of 192 wild populations of Atlantic salmon (*Salmo salar*) spanning the North Atlantic Ocean, including 142 from North America (*n* = 4993) and 50 from Europe (i.e., Norway, *n* = 806; Figure 1, Table S1). Samples of North American farmed salmon were collected from producers present in southern Newfoundland (*n* = 403) spanning a period from 2011 to 2018 (Table S1) and samples of Norwegian farmed salmon (*n* = 189). Samples of North American escaped farmed salmon (2013–2020) were collected from fish counting fences in rivers and as part of targeted recapture efforts following several escape events in southern Newfoundland and Nova Scotia (*n* = 289, Table S1). In all cases, fin clips were collected and preserved in 95% ethanol. For comparison, data from Bourret et al. (2013) encompassing 31 European locations are also included to provide broad representation of diversity in European wild salmon.

### 2.2 SNP genotyping

DNA was extracted using DNeasy Blood and Tissue or DNeasy 96 Blood and Tissue kits (Qiagen) following manufacturer's protocols. Concentration of extracted DNA was assessed using a Nanodrop spectrophotometer and by agarose gel visualization. DNA was standardized to a concentration of 15 ng/μl. A total of 6607 individuals were genotyped by Centre for Integrative Genetics (CIGENE) using a 220 K bi‐allelic single nucleotide polymorphism (SNP) Affymetrix Axiom array developed for Atlantic salmon as described in Barson et al. (2015). We retained a final dataset of 6491 individuals after excluding duplicate samples, and samples with aberrant genotypes indicating interspecific hybridization, or incomplete metadata. Genotype data were filtered for high quality SNPs based on their clustering patterns, retaining only SNPs with “polymorphic high” assignments and call rates >0.99. Subsequent filtering was performed using PLINK v 1.9 (Chang et al., 2015). SNPs were filtered for a minor allele frequency (MAF) cutoff of 0.01 across loci, resulting in total of 186,604 SNPs for downstream analyses.

### 2.3 Quantification of population structure and introgression in baseline wild populations

To quantify population structure in wild populations from Europe and North America, we first carried out model‐based clustering (Admixture 1.3.0, Alexander et al., 2009) on all 5799 wild individuals. We ran Admixture with 79,868 unlinked SNPs pruned using plink (i.e., 50 SNP windows, five SNP sliding windows, and *R* ^2^ threshold of 0.5), using K = 2. To produce a baseline population for quantifying wild population structure and comparison with farmed Atlantic salmon, we then screened all North American individuals and removed those with European *Q* values (i.e., estimates of European admixture) exceeding 5% (*n* = 25), indicating potential recent European introgression following Lehnert et al. (2019), who reported ~3% naturally occurring European ancestry in some North American populations. To quantify baseline population divergence between Europe and North America, we then carried out a second round of Admixture analysis with K = 2 using the 5774 baseline samples. We then conducted principal component analysis (PCA) in *pcadapt* (Luu et al., 2016) with K = 2 to separate individuals in multidimensional space using the full dataset of 186,604 SNPs and the complete North American and European baseline. Last, we calculated Weir and Cockerham's *F*_ST_ between Europe and North America in PLINK.

### 2.4 Quantification of population structure and introgression in farmed, farm escapes, and natural populations

Levels of European introgression in farmed and escaped farmed salmon were then quantified using two independent approaches. First, we used principal component analyses conducted in *pcadapt* to explore similarity between the farmed or escaped farmed individuals and the trans‐Atlantic (i.e., European and North American wild) baseline. Secondly, we estimated European and North American ancestry of farmed and escaped farmed individuals in separate runs of Admixture with K = 2 containing the European and North American baseline samples (5774) and farm (289) and escaped farmed (403) individuals. The PCA and Admixture analyses of farmed and escaped farmed salmon were also been repeated using a sample of Norwegian farmed salmon (*n* = 189) and a smaller SNP (*n* = 1910 SNPs) baseline (Bourret et al., 2013) containing broad European representation (Figure S2).

### 2.5 Genomic distribution of admixture

To quantify the presence, position, and size (or proportion of chromosome) of European ancestry tracts across the genomes of hybridized and introgressed individuals, we used PCAdmix v3 (Brisbin et al., 2012), which employs a principal‐component‐based algorithm to assign ancestry tracts along each chromosome using phased SNP genotypes of admixed individuals, compared against panels of samples with known ancestry. Phasing of SNP genotypes was done using Beagle 3.3 (Browning & Browning, 2007). For this analysis, two baseline groups (i.e., ancestral populations) of 50 individuals were selected from Europe and North America samples, which exhibited no evidence of introgression. We then ran PCAdmix separately on each chromosome in 20 SNP windows, without pruning for linkage, with ancestry assignments on six sets of 10 individuals (20 haplotypes), which displayed the highest European ancestry, grouped as: (1) North American individuals with no evidence of European introgression, (2) North American individuals from locations near aquaculture sites with evidence of European introgression (Figure S1), (3) North American individuals from locations with evidence of historical European introgression previously identified as a region of natural trans‐Atlantic secondary contact (Figure S1, Bradbury et al., 2015; Lehnert et al., 2019), (4) aquaculture salmon, (5) escaped farmed salmon, or (6) European salmon. To infer differences in the size of European ancestry tracts between groups, we calculated the mean and maximum size of continuous windows of European ancestry as a proportion of the total number of windows on each chromosome.

## 3 RESULTS

### 3.1 SNP genotyping and trans‐Atlantic differences

**FIGURE 2:**
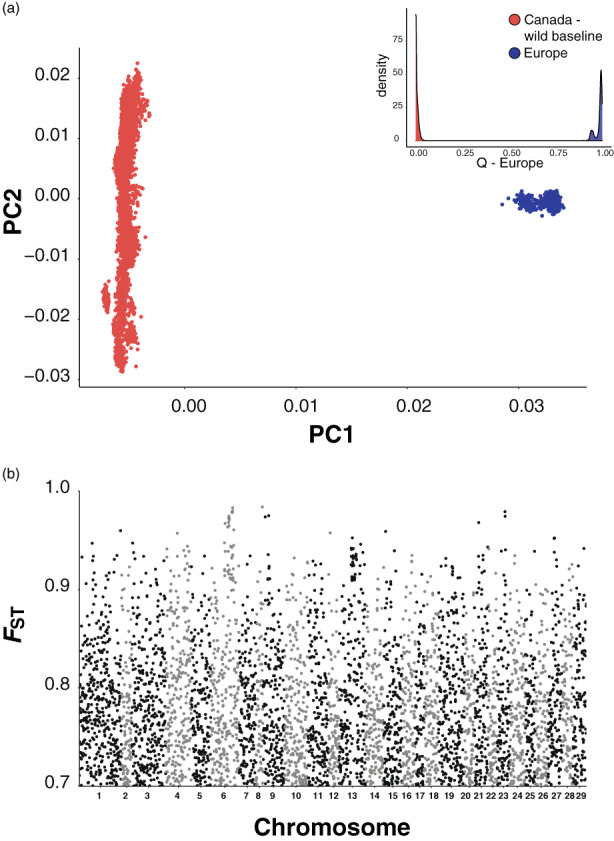
(a) Principal Component Analysis of Atlantic salmon using 186,597 SNPs highlighting trans‐Atlantic differences. Samples are colored by continent of origin, red for North America and blue for Europe; inset shows frequency distribution of European q‐values colored by group (79,868 unlinked SNPs). (b) Manhattan plot of locus‐specific FST of top 5000 SNPs most divergent between North America and Europe across the Atlantic salmon genome.

Initial PCA using the full dataset of 186,604 SNPs showed a clear separation of European and North American baseline individuals (Figure 2a) with PC1 explaining 34.97% of the variance. The magnitude of trans‐Atlantic differentiation was high (weighted *F*_ST_ = 0.436) and distributed across all chromosomes. The Manhattan plot of locus‐specific *F*_ST_ for the top 5000 SNPs, which exhibited *F*_ST_ greater than 0.7, showed high trans‐Atlantic divergence distributed evenly across the Atlantic salmon genome (Figure 2b).

### 3.2 Quantifying levels of introgression in wild and farmed populations

**FIGURE 3:**
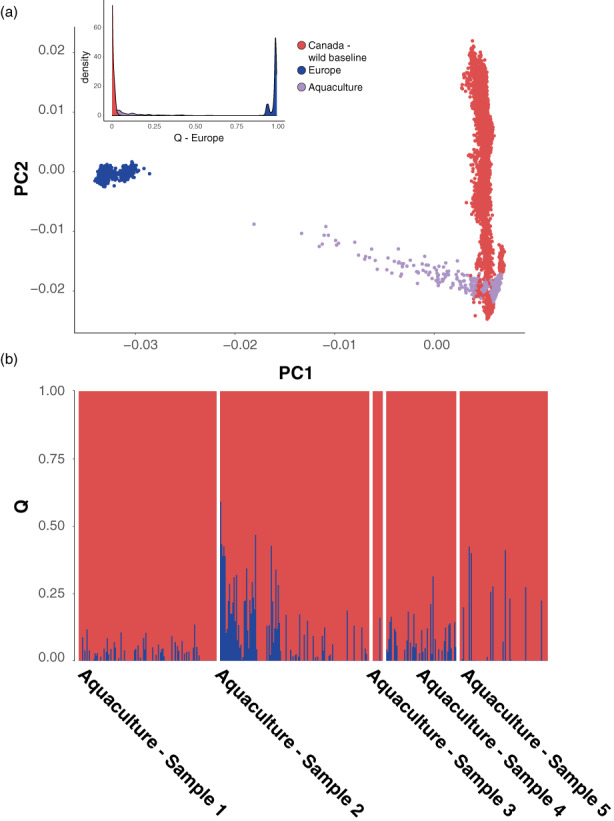
Estimates of European ancestry for North American aquaculture Atlantic salmon samples. (a) Principal Component Analysis for aquaculture salmon (purple) in comparison to North American (red) and European (blue) baselines, inset shows frequency distribution of European *q*‐values colored by group (see below); (b) European admixture (i.e. *Q*‐values) for five samples of North American aquaculture Atlantic salmon. See methods for details regarding the baseline samples used for comparison.

We assessed levels of European introgression in samples from five aquaculture cage sites in southern Newfoundland using both PCA and ADMIXTURE (Figure 3). PCA of the aquaculture samples showed evidence of European introgression in several aquaculture samples, with individuals intermediate between Europe and North America on PC1 detected (Figure 3a). The position of these intermediate PC1 samples on PC2 is consistent with the geographic region (i.e., Bay of Fundy) of origin of North American aquaculture salmon (Figure 3a). The magnitude of introgression was quantified using ADMIXTURE, which suggested that approximately 17% of the samples (70/403) were characterized by evidence of recent, significant European ancestry (>10% ancestry), with some individuals exceeding 40% European ancestry (Figure 3b). The degree of European ancestry among aquaculture samples varied significantly (mean Q = 0.0178–0.0829, − Kruskwal–Wallis test, Chi‐squared = 36.06, *df* = 4, *p* = 2.53 × 10^−7^) among the samples. Repeating both the Admixture and PCA analyses of the North American aquaculture samples with the additional baselines (i.e., Norwegian farmed Atlantic salmon, and the additional European SNP baseline [Figure S2, Bourret et al., 2013]) produced results consistent with those produced using the Norwegian genomic baseline (see Figures S3 and S4).

**FIGURE 4:**
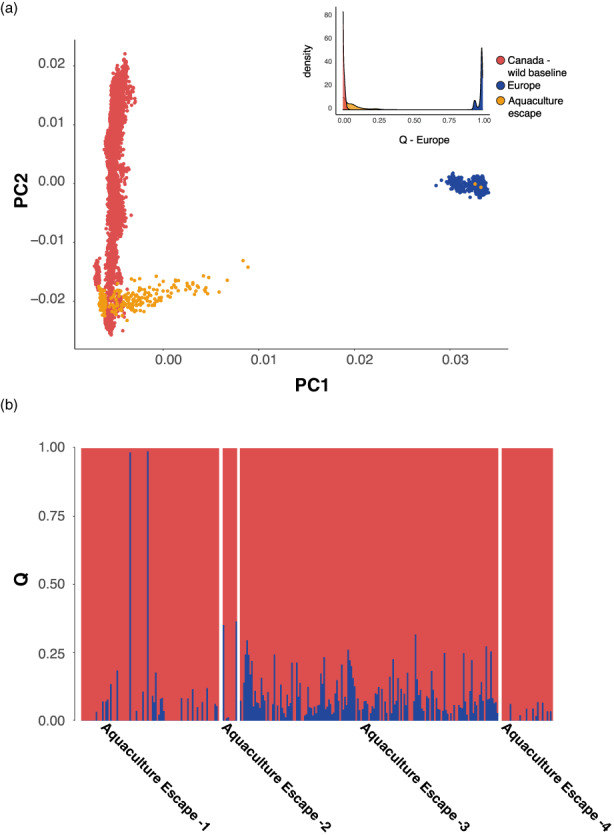
Estimates of European ancestry for North American escaped farmed Atlantic salmon samples. (a) Principal Component Analysis for aquaculture salmon (orange) in comparison to North American (red) and European (blue) baselines, inset shows frequency distribution of European *q*‐values colored by group (see below); (b) European admixture (i.e. *Q*‐values) for the four samples of North American escaped farmed Atlantic salmon. See methods for details regarding the baseline samples used for comparison.

Analysis of aquaculture escapee samples also indicated significant levels of European ancestry (Figure 4). Again, the PCA revealed a subset of individuals intermediate between Europe and North America on PC1 and similarity with the source region of origin for North American aquaculture salmon on PC2 (Figure 4a). The level of admixture detected varied significantly among samples, with aquaculture escape sample three displaying the most consistent levels of European ancestry (Figure 4b). The ADMIXTURE analysis suggested significant introgression, and at levels greater than in cage sites (mean Q Europe cage = 0.048, escape = 0.0621, Mann–Whitney test, *p* = 1.295 × 10^−5^, W = 47,680). We also detected two individuals that displayed >99% European ancestry in the ADMIXTURE analysis (Figure 4b). Once again, repeating both the PCA and Admixture analyses of the North American escaped farmed salmon using the additional baselines (i.e., Norwegian farmed Atlantic salmon and the expanded European SNP baseline [Figure S2, Bourret et al., 2013]) produced both PCA and Admixture results consistent with those produced using the Norwegian genomic baseline (Figures S3 and S4).

**FIGURE 5:**
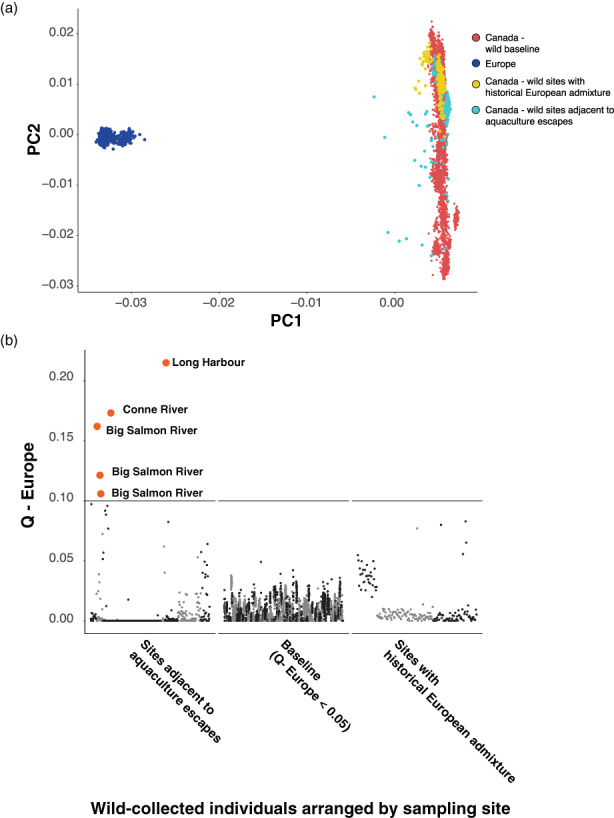
Estimates of European ancestry for wild collected North American Atlantic salmon samples. (a) Principal Component Analysis for wild collected salmon (yellow and light blue) in comparison to North American (red) and European (dark blue) baselines, see methods for group details; (b) European admixture values (i.e., *Q*‐values) for North American Atlantic salmon samples collected in the wild. Samples exceeding 10% European admixture are colored red. Wild sites are classified as (1) North American locations near aquaculture sites with evidence of European introgression; (2) North American individuals with no evidence of European introgression; (3) North American wild locations with evidence of historical European introgression (see Section 2).

Finally, juvenile wild salmon sampled throughout Atlantic Canada previously removed from the baseline due to levels of European admixture exceeding 5% were screened for European ancestry (Figure 5). These individuals were categorized geographically as either occurring near aquaculture cage sites where hybridization with escapees had previously been reported (Sylvester et al., 2019; Wringe et al., 2018) or to the east where no escapees have been detected but a region of postglacial trans‐Atlantic secondary contact has been identified (Bradbury et al., 2015; Lehnert et al., 2019). The PCA revealed samples that lay between continents both at sites near aquaculture cages and sites in the region of secondary contact. However, samples collected near the cage sites occurred closer to Europe on PC1 than samples near the region of secondary contact, suggesting more recent introgression. ADMIXTURE analysis indicated that the highest levels of European ancestry (>10%) detected in the wild collected juveniles (Figure 5b) were from three locations (Conne River, Long Harbour River, and Big Salmon River) all of which have had previous evidence of both escapees and/or hybridization with escapees. In contrast, genome‐wide admixture estimates from sites distant to salmon aquaculture, even across the region of secondary contact, did not exceed 10%. However, elevated admixture values (i.e., 3%–9%) were detected at several locations across the secondary contact zone.

### 3.3 Genomic distribution of admixture

**FIGURE 6:**
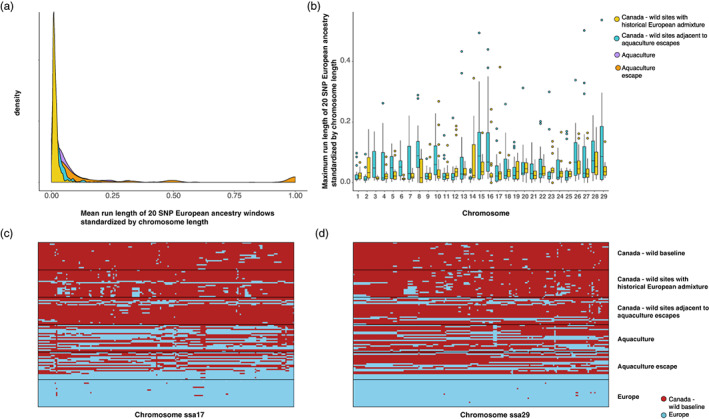
The size and distribution of European ancestry tracts across the genomes of aquaculture and wild collected samples. (a) frequency distribution of mean run of European ancestry standardized by chromosome length for escapees (orange), aquaculture samples (purple), wild collected juveniles near aquaculture cage sites (blue), and finally wild collected juveniles from the zone of secondary contact (yellow); (b) Maximum run length of European ancestry standardized by chromosome length; distribution of European ancestry across chromosome (c) ssa17 and (d) ssa29 by group. Each group consists of 10 individuals with the highest European ancestry from the available samples of each category but see methods for further group descriptions and details.

We used PCAdmix to characterize the proportion, sizes, and distribution of European ancestry tracts across the genomes of aquaculture and wild samples (Figure 6). The genome‐wide average proportion of a chromosome that was characterized by European ancestry was highest in escapee (0.205) and aquaculture (0.099) samples, followed by wild collected juveniles near aquaculture cage sites (0.024), and finally wild collected juveniles from the zone of secondary contact (0.0163; Figure 6a). Comparison of average ancestry tract size across all chromosomes indicated significantly greater average European ancestry tract size among individuals from sites near recent aquaculture cages and escapee detections compared with individuals collected in the zone of secondary contact (W = 147,944, *p* < 0.0005). Similarly, maximum European ancestry tract size between these groups was also generally larger in individuals collected near aquaculture cage locations than across the region of secondary contact (Figure 6b). For the majority (23/29) of chromosomes, the largest European ancestry tracts in individuals sampled in the wild were found in individuals collected near cage sites (Figure 6b). The maximum value for European ancestry tract length in samples from the region of secondary contact was observed at chromosome ssa17 (max European proportion = 0.381 Figure 6c), and the maximum value for European ancestry tract length in aquaculture adjacent locations was observed at chromosome ssa29 (max European proportion = 0.536, Figure 6d).

## 4 DISCUSSION

Hybridization and introgression between wild and domestic individuals have been shown to be common to the domestication process across a diverse range of taxa (Liu et al., 2021; Wu et al., 2018). Although introgression between divergent lineages has been used to increase production in captive stocks, it can also increase maladaptation of wild individuals to a given natural environment (Baskett et al., 2013; Fleming, 1995). We found that some North American farmed Atlantic salmon and farm escapees collected between 2011 and 2020 were characterized by moderate to high levels of European ancestry, consistent with the presence of pure European individuals or hybrids in Canadian breeding programs during that period. Moreover, the analysis of individuals collected in the wild, including across a region of natural historical trans‐Atlantic secondary contact, indicated elevated European ancestry and larger European ancestry blocks in samples from rivers near aquaculture cage sites suggesting that some of these aquaculture individuals have escaped and hybridized with wild Atlantic salmon. The results build on earlier work that reported the presence of European microsatellite alleles in escaped farmed salmon collected in the Bay of Fundy (DFO, 2018b; O'Reilly et al., 2006) and an evaluation of the risk of using diploid European domestic salmon in Atlantic Canada (DFO, 2013). Ultimately, this work suggests that trans‐Atlantic hybridization of Atlantic salmon has been a persistent part of domestication programs in North America, elevating the potential impact and uncertainty regarding the risk posed by escapees to wild and often at‐risk salmon populations.

The process of domestication across plant and animal groups has been shown to commonly include interbreeding among source populations, species, or strains to maximize production (Arnold, 2004; Purugganan, 2019; Walker, 2013). Our results suggest that North American farmed salmon and escapees sampled within Atlantic Canada included individuals that were either pure European, North American/European hybrids, or introgressed individuals. These observations are consistent with studies conducted over the last 15 years reporting the presence of European introgression into North American domestic salmon (i.e., Saint. John River strain) and escapees. These include recent experimental genomic studies using North American farmed salmon, which reported prescreening for European admixture prior to further analysis (Holborn et al., 2018, 2020; Liu et al., 2017). Indeed, one study reported having to exclude ~27% of farmed fish analyzed for levels of European admixture exceeding 5% (Liu et al., 2017). Similarly, O'Reilly et al. (2006) detected European ancestry in escapees in the Bay of Fundy using microsatellite loci and reported that escaped parr, smolts, and adults all had European‐type alleles. Taken in this context, our results represent the most recent and extensive addition to almost two decades of work concluding that even though diploid European salmon have never been approved for use in Canada (O'Reilly et al., 2006; Porter et al., 1998), European hybridization and introgression into farmed salmon in Atlantic Canada has occurred and persists. In contrast to previous work, we demonstrate for the first time the presence of both high levels of European admixture and pure European individuals in samples of North American farmed salmon in Atlantic Canada.

Atlantic salmon escapes from net‐pen aquaculture continue to be frequent occurrences (Diserud et al., 2019; Glover et al., 2019; Keyser et al., 2018). Escapees have been shown to breed with wild salmon (Glover et al., 2017), and consequently, escaped farm salmon have been identified as a significant threat to the persistence of salmon in the wild (Forseth et al., 2017). Our observations of significant European admixture in captive and escaped aquaculture salmon strongly suggest that individuals with significant European admixture have escaped and have reproduced in the wild. Our results showing elevated European ancestry (>10%) in juveniles collected from rivers near salmon aquaculture cage sites unequivocally support the hypothesis that this occurred. Several of these locations displaying elevated European ancestry both in southern Newfoundland and in the Bay of Fundy have previously been identified as rivers where hybridization between wild and farm escaped salmon have occurred (DFO, 2018b; O'Reilly et al., 2006; Sylvester et al., 2019). For example, Wringe et al. (2018) reported extensive hybridization in southern Newfoundland following an escape event in 2013, which included the detection of first‐generation hybrids at Conne River, and Long Harbour River where we report elevated levels of European admixture. Following on this, work has continued to detect the presence of farmed‐wild hybrids at these locations. In the Bay of Fundy, previous work has also suggested the presence of escapees (Morris et al., 2008) and that hybridization with escapees bearing European ancestry has occurred (DFO, 2008, 2018b; O'Reilly et al., 2006).

Interestingly, introgression between North American and European salmon has also occurred historically, likely following postglacial recolonization of some northern areas (King et al., 2007; Lehnert et al., 2019). Several studies have identified regions of trans‐Atlantic secondary contact both in North America and in northern Europe (King et al., 2007; Rougemont & Bernatchez, 2018). In Atlantic Canada, existing genomic evidence suggests that secondary contact has occurred in southeastern Newfoundland and in Labrador (Bradbury et al., 2015; Sylvester et al., 2018; Watson et al., 2022). Following on this previous work, our results indicate evidence of elevated European admixture at several locations in southeastern Newfoundland across this previously identified region of secondary contact. Admittedly, this naturally occurring European admixture could reduce the power to resolve aquaculture escapee associated European introgression into wild populations in the region of secondary contact. In this region, our results suggest that both the magnitude and genomic distribution of European admixture (see below) may be informative. However, it is also important to note that areas near cage sites where we observed elevated admixture are outside the region of secondary contact and that no escapees have been reported in rivers where secondary contact has been detected. It is nonetheless interesting that we observed elevated levels of postglacial European admixture between 5% and 9% across a few locations in the region of secondary contact. Previous work suggests that the European lineage in Newfoundland was isolated from Europe ~18,000–19,000 years ago (Bradbury et al., 2015). As such, it is perhaps surprising to see these levels of European admixture persist to the present day. Salmon populations in the region are generally small and characterized by high levels of isolation (Bradbury et al., 2014; COSEWIC, 2010; DFO, 2012, 2020). Also, it is worth noting that there is evidence of selection associated with these genomic regions of introgression (Watson et al., 2022). Both factors (i.e., limited gene flow and selection) may have facilitated the retention of European elevated ancestry across individuals in the region of secondary contact.

Previous work has demonstrated that the length of ancestry tracts can be used to infer the time since introgression or the spatial scale of dispersal (Duranton et al., 2019; Leitwein et al., 2018, 2020). With each generation following a migration event, ancestry tracts are progressively shortened by recombination; therefore, longer ancestry tracts may be indicative of more recent migration compared with shorter ones (Leitwein et al., 2018; Liang & Nielsen, 2014; Pool & Nielsen, 2009). Our results suggest that European ancestry tracts were generally larger in individuals collected near aquaculture cage sites than across the region of secondary contact, consistent with recent European introgression from aquaculture escapees. Admittedly, this conclusion makes several assumptions. First, it assumes that there is sufficient divergence among lineages to allow accurate delineation of ancestry tracts. For trans‐Atlantic differences in Atlantic salmon, which have been hypothesized to represent distinct subspecies, levels of genome wide divergence are likely more than sufficient to accurately resolve the genomic distribution of admixture (Lehnert et al., 2020). The exception to this generalization may be genomic regions harboring structural variants associated with post‐glacial secondary contact between European and North American lineages, such as the chromosomal trans‐location previously identified between chromosome Ssa01 and Ssa23 (Lehnert et al., 2019). Second, the interpretation assumes that ancestry tract lengths are not influenced by selection. It is possible that selection favoring genomic regions containing European admixture may be present. Watson et al. (2022) report evidence of environmental associations with genomic regions associated with European introgression, in this case the European Ssa01/23 karyotype. It is possible that selection has played a role in maintaining larger European ancestry tract lengths in some locations across the region of secondary contact, and this may explain locations with 6%–9% European ancestry. Finally, the analysis assumes that the density of markers across the genome is sufficient to accurately resolve the length of ancestry tracts. In this context given we have ~186 K SNPs, and we are evaluating evidence of recent hybridization and introgression, it seems unlikely this is a concern; however, whole genome resequencing on a subset of individuals could be used to further evaluate accuracy here. It is also worth noting that our primary European genomic baseline comprises Norwegian salmon populations and potentially not representative more broadly of the European Atlantic range. However, given (1) the magnitude of trans‐Atlantic differentiation observed and reported previously (Bourret et al., 2013; Jeffery et al., 2018; King et al., 2001; Lehnert et al., 2020) and (2) the fact that the European aquaculture salmon used in eastern North America have been thought to have predominately originated in Norway, we expect any potential bias to be negligible. We do, however, go further and demonstrate through the use of both Norwegian aquaculture samples and a secondary SNP baseline encompassing the three large European phylogenetic groups that our conclusions of European ancestry in North American farmed, farm escaped, and wild salmon are robust to the choice of European baseline utilized.

Significant uncertainty remains as to the magnitude of negative impacts of European farmed escaped salmon or their offspring on wild salmon populations in North America (DFO, 2013, 2016). Differences between European and North American Atlantic salmon associated with allele frequencies (Bourret et al., 2013; Jeffery et al., 2018; King et al., 2001; Lehnert et al., 2020) and karyotypic differences (Brenna‐Hansen et al., 2012; Lehnert et al., 2019) have been long recognized. These trans‐Atlantic genetic differences have been associated with metabolic, developmental, immune, and neural processes and as such may represent adaptive divergence (Lehnert et al., 2020). This hypothesis of significant adaptive differences has been supported by previous genomic and experimental work. Cauwelier et al. (2012) examined the viability of crosses of Scottish and Canadian salmon and reported complete lack of viability of Canadian backcrosses suggesting asymmetric outbreeding depression. Furthermore, Bradbury et al. (2015) reported asymmetry in the distribution of admixture values for salmon sampled across the region of secondary contact with values skewed toward the European type. In addition to natural trans‐Atlantic differences, European salmon have also been subjected to a longer period of domestication selection compared with North American Atlantic salmon (Glover et al., 2017). Ultimately, the degree of maladaptation associated with trans‐Atlantic movement remains uncertain. It does, however, seem reasonable to conclude that due to trans‐Atlantic differentiation and an increased period of domestication for European salmon, the degree of maladaptation may be elevated for European farmed salmon in North America compared with North American farmed salmon.

The potential for significant maladaptation of European salmon in North America is consistent with previous management decisions regarding their usage in both the United States and Canada (Baum, 1998; DFO, 2013, 2016, 2018a; NRC[USA], 2004). The enforcement of such management decisions requires accurate screening tools to resolve levels of European admixture. Our results suggest that large SNP panels represent an effective approach and provide the resolution to detect and quantify European admixture within aquaculture salmon and wild populations. It seems likely that smaller targeted panels of the SNPs examined could prove useful screening tools particularly for aquaculture salmon. However, our results also suggest that the power to identify aquaculture‐associated European admixture in wild populations will be region‐specific and require evaluations of the genomic distribution of admixture in regions characterized by postglacial trans‐Atlantic secondary contact such as in southeastern Newfoundland. Overall, it seems based on genome‐wide values of European admixture, our results suggest that 10% may be a robust threshold for screening for natural introgression though lower values may be achievable in regions with no history of post‐glacial secondary contact (e.g., Bay of Fundy).

## 5 CONCLUSIONS

The importance of domesticated marine and freshwater fish species is poised to increase in the coming years, as is the conservation concern regarding the status of many of these species in the wild. Our results provide evidence that even though diploid European salmon have never been approved for use in Canada, individuals of full and partial European ancestry have been used over the last decade, and that some of these individuals have escaped and hybridized in the wild. Our use of trans‐Atlantic sampling and genomic data in this context has allowed accurate estimation of European admixture and of European ancestry tract length in aquaculture salmon and escapees. This approach likely significantly improves both the accuracy and resolution over previous microsatellite‐based methods, particularly as aquaculture expands into regions of postglacial trans‐Atlantic secondary contact. Future targeted amplicon or SNP panel development based on these results is likely to allow routine screening for European admixture of aquaculture or hatchery samples and inform robust thresholds for comparison.

## CONFLICTS OF INTEREST

The authors declare no conflict of interest.

## Supporting information


**Table S1.** Baseline, escapee, and aquaculture samples, geographic position (if applicable), and sample sizes.
**Figure S1.** Map of locations where juvenile salmon with elevated European ancestry were detected including both locations near aquaculture cages (blue) and those distant from cages but with evidence for naturally occurring post‐glacial secondary contact between North American and European salmon (yellow) as well as aquaculture site locations (purple).
**Figure S2**. Map of European baseline locations from Bourret et al. (2013) included for comparison (see Figures S3 and S4).
**Figure S3.** Estimates of European ancestry for North American aquaculture, escapee, and wild collected Atlantic salmon samples using baseline North American and European Atlantic salmon data from Table S1 as well as European and North American SNP data from Bourret et al. 2013 with distinct European groupings indicated (Figure S2) using 1910 SNPs.
**Figure S4.** Estimates of European ancestry for North American aquaculture, escapee, and wild collected Atlantic salmon samples using baseline North American (Table S1) and Norwegian aquaculture salmon.

## Data Availability

The SNP data for Canadian populations are taken from Lehnert et al. (2020), the SNP data for aquaculture individuals will be accessible via DYRAD (https://doi.org//), and the SNP data for Norwegian populations are taken from Barson et al. (2015). Additional baseline European salmon SNP data used in the supplemental analysis are taken from Bourret et al. (2013).
