# Supplementary material for: Genomic evidence of recent European introgression into North American farmed and wild Atlantic salmon
Source: Evol Appl. 2022 Aug 24;15(9):1436–48. doi: 10.1111/eva.13454 (PMC9488674; doi:10.1111/eva.13454)
Supplement: Supplementary file 1 — Table S1. Baseline, escapee, and aquaculture samples, geographic position (if applicable), and sample sizes. Figure S1. Map of locations where juvenile salmon with elevated European ancestry were detected including both locations near aquaculture cages (blue) and those distant from cages but with evidence for naturally occurring post‐glacial secondary contact between North American and European salmon (yellow) as well as aquaculture site locations (purple). Figure S2. Map of European baseline locations from Bourret et al. (2013) included for comparison (see Figures S3 and S4). Figure S3. Estimates of European ancestry for North American aquaculture, escapee, and wild collected Atlantic salmon samples using baseline North American and European Atlantic salmon data from Table S1 as well as European and North American SNP data from Bourret et al. 2013 with distinct European groupings indicated (Figure S2) using 1910 SNPs. Figure S4. Estimates of European ancestry for North American aquaculture, escapee, and wild collected Atlantic salmon samples using baseline North American (Table S1) and Norwegian aquaculture salmon. [file EVA-15-1436-s001.docx]

**Supplemental Information**

**for**

**Genomic evidence of European introgression into North American farmed and Wild Atlantic Salmon**

Table S1. Baseline, escapee and aquaculture samples, geographic position (if applicable) and sample sizes. See text for details on data collection. See Bourret et al. (2013) for additional baseline samples used in supplemental analysis in Figures S2-S4.

|  |  |  |  |  |  |
| --- | --- | --- | --- | --- | --- |
| \| Site \| FID \| Group \| Province / Region \| Lat \| Lon \| n \| \| --- \| --- \| --- \| --- \| --- \| --- \| --- \| \| Aquaculture Sample 1 \| AQUA1 \| Aquaculture \| Newfoundland \|  \|  \| 122 \| \| Aquaculture Sample 2 \| AQUA2 \| Aquaculture \| Newfoundland \|  \|  \| 9 \| \| Aquaculture Sample 3 \| AQUA3 \| Aquaculture \| Newfoundland \|  \|  \| 62 \| \| Aquaculture Sample 4 \| AQUA4 \| Aquaculture \| Newfoundland \|  \|  \| 78 \| \| Aquaculture Sample 5 \| AQUA5 \| Aquaculture \| Newfoundland \|  \|  \| 132 \| \| Aquaculture Escape Sample 1 \| ESC1 \| Escapee \| Newfoundland \|  \|  \| 86 \| \| Aquaculture Escape Sample 2 \| ESC2 \| Escapee \| Newfoundland \|  \|  \| 9 \| \| Aquaculture Escape Sample 3 \| ESC3 \| Escapee \| Nova Scotia \|  \|  \| 1 \| \| Aquaculture Escape Sample 4 \| ESC4 \| Escapee \| Newfoundland \|  \|  \| 161 \| \| Aquaculture Escape Sample 5 \| ESC5 \| Escapee \| Newfoundland \|  \|  \| 32 \| \| Jupiter River \| JU \| North America \| Anticosti \| 49.47 \| -63.58 \| 28 \| \| Big River \| BIG \| North America \| Labrador \| 54.84 \| -58.94 \| 28 \| \| Cape Caribou River \| CB \| North America \| Labrador \| 53.62 \| -60.42 \| 21 \| \| Caroline River \| CL \| North America \| Labrador \| 53.25 \| -60.42 \| 18 \| \| Crooked River \| CR \| North America \| Labrador \| 53.87 \| -60.83 \| 21 \| \| Eagle River \| EA \| North America \| Labrador \| 53.53 \| -57.47 \| 21 \| \| English River \| ENG \| North America \| Labrador \| 54.97 \| -59.75 \| 27 \| \| Forteau River \| FO \| North America \| Labrador \| 51.48 \| -56.94 \| 21 \| \| Hunt River \| H \| North America \| Labrador \| 55.57 \| -60.67 \| 19 \| \| Kenamu River \| KE \| North America \| Labrador \| 53.48 \| -59.91 \| 22 \| \| L'anseau Loup River \| LL \| North America \| Labrador \| 51.53 \| -56.82 \| 22 \| \| Main Brook \| MNB \| North America \| Labrador \| 54.24 \| -57.87 \| 21 \| \| Mulligan River \| MU \| North America \| Labrador \| 53.87 \| -60.09 \| 22 \| \| Paradise River \| PA \| North America \| Labrador \| 53.42 \| -57.25 \| 19 \| \| Peters River \| PR \| North America \| Labrador \| 53.34 \| -60.71 \| 21 \| \| Red Wine River \| RW \| North America \| Labrador \| 53.93 \| -61.00 \| 22 \| \| Sand Hill River \| SH \| North America \| Labrador \| 53.57 \| -56.35 \| 19 \| \| Sebaskachu River \| SK \| North America \| Labrador \| 53.79 \| -60.14 \| 22 \| \| St Charles River \| CHR \| North America \| Labrador \| 52.23 \| -55.84 \| 27 \| \| Susan River \| SR \| North America \| Labrador \| 53.74 \| -61.04 \| 22 \| \| Big East River \| BER \| North America \| Labrador \| 50.63 \| -57.17 \| 27 \| \| Narraguagus River \| NGR \| North America \| Maine / USA \| 44.52 \| -67.86 \| 21 \| \| Sheepscot River \| SHP \| North America \| Maine / USA \| 43.91 \| -69.67 \| 20 \| \| Kedgwick River \| KED \| North America \| New Brunswick \| 47.91 \| -67.91 \| 15 \| \| Kouchibouguac River \| KOU \| North America \| New Brunswick \| 46.74 \| -65.20 \| 31 \| \| Miramichi-Upper Northwest \| MUN \| North America \| New Brunswick \| 47.17 \| -65.94 \| 24 \| \| Miramichi-Upper Southwest \| MSW \| North America \| New Brunswick \| 46.55 \| -66.04 \| 23 \| \| Nashwaak River \| NSH \| North America \| New Brunswick \| 45.96 \| -66.62 \| 19 \| \| Patapedia River \| PAT \| North America \| New Brunswick \| 47.86 \| -67.39 \| 24 \| \| Richibucto River \| RIC \| North America \| New Brunswick \| 46.36 \| -65.15 \| 31 \| \| Upsalquitch River \| UPS \| North America \| New Brunswick \| 47.57 \| -66.54 \| 28 \| \| Avondale River \| AVR \| North America \| Newfoundland \| 47.39 \| -53.23 \| 29 \| \| Bay de L'Eau River \| BDL \| North America \| Newfoundland \| 47.51 \| -54.73 \| 91 \| \| Bay du Nord \| BDN \| North America \| Newfoundland \| 47.73 \| -55.44 \| 20 \| \| Bear Cove Brook \| BCB \| North America \| Newfoundland \| 47.67 \| -59.30 \| 29 \| \| Beaver Brook \| BVB \| North America \| Newfoundland \| 50.90 \| -56.15 \| 29 \| \| Big Salmonier Brook \| BSA \| North America \| Newfoundland \| 47.06 \| -55.22 \| 81 \| \| Black River \| BLA \| North America \| Newfoundland \| 47.89 \| -54.17 \| 82 \| \| Branch River \| BRA \| North America \| Newfoundland \| 46.89 \| -53.97 \| 68 \| \| Campbellton River \| CMP \| North America \| Newfoundland \| 49.28 \| -54.93 \| 25 \| \| Cape Roger Brook \| CRB \| North America \| Newfoundland \| 47.44 \| -54.69 \| 56 \| \| Cinq Cerf River \| CCR \| North America \| Newfoundland \| 47.70 \| -58.15 \| 30 \| \| Collinet River \| CLR \| North America \| Newfoundland \| 47.22 \| -53.55 \| 30 \| \| Come By Chance River \| CBC \| North America \| Newfoundland \| 47.97 \| -53.96 \| 61 \| \| Conne River \| CNR \| North America \| Newfoundland \| 47.91 \| -55.70 \| 209 \| \| Couteau Brook \| CBR \| North America \| Newfoundland \| 47.75 \| -58.03 \| 30 \| \| Cuslett Brook \| CUS \| North America \| Newfoundland \| 46.96 \| -54.16 \| 87 \| \| Dollards Brook \| DLR \| North America \| Newfoundland \| 48.02 \| -56.57 \| 26 \| \| East Bay Brook \| EBB \| North America \| Newfoundland \| 47.77 \| -58.25 \| 29 \| \| Fair Haven Brook \| FHB \| North America \| Newfoundland \| 47.54 \| -53.89 \| 103 \| \| Farmer's Brook \| FRM \| North America \| Newfoundland \| 47.67 \| -58.48 \| 30 \| \| Flat Bay Brook \| FLB \| North America \| Newfoundland \| 48.41 \| -58.58 \| 24 \| \| Garia Brook \| GBK \| North America \| Newfoundland \| 47.75 \| -58.53 \| 29 \| \| Garnish River \| GAR \| North America \| Newfoundland \| 47.23 \| -55.35 \| 22 \| \| Grand Bank Brook 2019 (Young of Year) \| GBB1 \| North America \| Newfoundland \| 47.09 \| -55.76 \| 28 \| \| Grand Bay \| GBR \| North America \| Newfoundland \| 47.63 \| -59.13 \| 30 \| \| Grand Codroy River \| COD \| North America \| Newfoundland \| 47.84 \| -59.20 \| 57 \| \| Grandy Brook (Burgeo) \| GND \| North America \| Newfoundland \| 47.89 \| -57.72 \| 29 \| \| Grandy Brook (Burnt Islands) \| GNB \| North America \| Newfoundland \| 47.63 \| -58.83 \| 30 \| \| Great Barasway Brook \| GBW \| North America \| Newfoundland \| 47.12 \| -54.06 \| 89 \| \| Great Rattling Brook-Exploits \| GRB \| North America \| Newfoundland \| 49.62 \| -56.17 \| 26 \| \| Grey River \| GRR \| North America \| Newfoundland \| 47.77 \| -56.93 \| 30 \| \| Humber River \| TYB \| North America \| Newfoundland \| 49.55 \| -57.10 \| 29 \| \| Isle aux Morts River \| IAM \| North America \| Newfoundland \| 47.59 \| -59.01 \| 57 \| \| La Poile River \| LPR \| North America \| Newfoundland \| 47.87 \| -58.24 \| 29 \| \| Lamaline River 2020 (Young of Year) \| LMS2020 \| North America \| Newfoundland \| 46.88 \| -55.78 \| 28 \| \| Lance River \| LAN \| North America \| Newfoundland \| 46.82 \| -54.07 \| 9 \| \| Lawn River \| LWN \| North America \| Newfoundland \| 46.95 \| -55.54 \| 80 \| \| Little Codroy \| LCO \| North America \| Newfoundland \| 47.83 \| -59.15 \| 27 \| \| Little Barasway Brook \| LBB \| North America \| Newfoundland \| 47.18 \| -54.03 \| 15 \| \| Little Salmonier \| LSR \| North America \| Newfoundland \| 47.07 \| -55.18 \| 17 \| \| Lomond River Bond Bay Pond \| LRBB \| North America \| Newfoundland \| 49.36 \| -57.65 \| 23 \| \| Lomond River East Branch \| LREB \| North America \| Newfoundland \| 49.40 \| 57.61 \| 23 \| \| Long Harbour \| LHR \| North America \| Newfoundland \| 47.82 \| -54.94 \| 18 \| \| Mobile River \| MOB \| North America \| Newfoundland \| 47.25 \| -52.84 \| 29 \| \| Nonsuch River \| NON \| North America \| Newfoundland \| 47.45 \| -54.64 \| 91 \| \| North Harbour River \| NHR \| North America \| Newfoundland \| 47.21 \| -53.62 \| 30 \| \| North Brook Trepassy \| NBT \| North America \| Newfoundland \| 46.74 \| -53.36 \| 25 \| \| Northeast Brook 2019(Young of Year) \| NEB19_YOY \| North America \| Newfoundland \| 47.73 \| -55.36 \| 28 \| \| Northeast Placentia River \| NPR \| North America \| Newfoundland \| 47.29 \| -53.80 \| 81 \| \| Northeast Placentia River \| NRP \| North America \| Newfoundland \| 47.29 \| -53.80 \| 28 \| \| North Harbour River \| NHR \| North America \| Newfoundland \| 47.92 \| -54.03 \| 87 \| \| Northwest Brook (MortierBay) \| NMB \| North America \| Newfoundland \| 47.17 \| -55.32 \| 87 \| \| Piercey's Brook \| PBR \| North America \| Newfoundland \| 46.88 \| -55.86 \| 83 \| \| Pipers Hole River \| PHR \| North America \| Newfoundland \| 47.93 \| -54.27 \| 87 \| \| Red Harbour River East \| RHA \| North America \| Newfoundland \| 47.33 \| -54.99 \| 91 \| \| Red Harbour River West \| RHW \| North America \| Newfoundland \| 47.30 \| -55.02 \| 75 \| \| Renews River \| RNR \| North America \| Newfoundland \| 46.94 \| -52.96 \| 30 \| \| Rushoon River \| RUS \| North America \| Newfoundland \| 47.37 \| -54.92 \| 84 \| \| Salmonier River \| SLR \| North America \| Newfoundland \| 47.20 \| -53.37 \| 30 \| \| Sandy Harbour River \| SHA \| North America \| Newfoundland \| 47.71 \| -54.36 \| 74 \| \| Ship Harbour Brook \| SHI \| North America \| Newfoundland \| 47.35 \| -53.87 \| 82 \| \| Simms Brook 2020 (Young of Year) \| SIM \| North America \| Newfoundland \| 47.67 \| -55.48 \| 28 \| \| Simms Brook \| SIM \| North America \| Newfoundland \| 47.67 \| -55.48 \| 8 \| \| Southeast Placentia River \| SPR \| North America \| Newfoundland \| 47.23 \| -53.88 \| 96 \| \| Tailrace Brook 2020 (Young of Year) \| TRB \| North America \| Newfoundland \| 48.01 \| -55.79 \| 28 \| \| Taylor Bay Brook (Burin Peninsula) \| TBR \| North America \| Newfoundland \| 46.88 \| -55.71 \| 80 \| \| Terenceville Brook 2020 (Young of Year) \| TEB \| North America \| Newfoundland \| 47.70 \| -54.65 \| 3 \| \| Terra Nova River \| TNR \| North America \| Newfoundland \| 48.67 \| -54.00 \| 29 \| \| Tides Brook \| TDS \| North America \| Newfoundland \| 47.13 \| -55.26 \| 68 \| \| Trout River Eastern Brook \| TREB \| North America \| Newfoundland \| 49.37 \| -57.96 \| 22 \| \| Trout River Feeder \| TRF \| North America \| Newfoundland \| 49.58 \| -57.90 \| 29 \| \| Trout River \| TR \| North America \| Newfoundland \| 49.65 \| -57.76 \| 22 \| \| Trout River Narrows Brook \| TRNB \| North America \| Newfoundland \| 49.43 \| -58.08 \| 3 \| \| Trout River Western Brook \| TRWB \| North America \| Newfoundland \| 49.36 \| -57.96 \| 13 \| \| Western Brook Stag Brook \| WBSB \| North America \| Newfoundland \| 49.75 \| -57.87 \| 28 \| \| Western Cove Brook (Chapel Arm) \| WCB \| North America \| Newfoundland \| 47.51 \| -53.68 \| 17 \| \| Western Arm \| WAB \| North America \| Newfoundland \| 51.19 \| -56.76 \| 18 \| \| White Bear River \| WBR \| North America \| Newfoundland \| 47.87 \| -57.28 \| 30 \| \| Big Salmon \| BSR \| North America \| Nova Scotia \| 45.42 \| -65.41 \| 22 \| \| Baddeck \| BAD \| North America \| Nova Scotia \| 46.10 \| -60.84 \| 28 \| \| Cheticamp River \| CHT \| North America \| Nova Scotia \| 46.64 \| -60.95 \| 12 \| \| Clyburne River \| CLY \| North America \| Nova Scotia \| 46.66 \| -60.41 \| 28 \| \| East River Pictou \| PIE \| North America \| Nova Scotia \| 45.54 \| -62.88 \| 23 \| \| Gaspereau River \| GAK \| North America \| Nova Scotia \| 45.06 \| -64.38 \| 26 \| \| Graham River \| JGC \| North America \| Nova Scotia \| 45.86 \| -61.49 \| 11 \| \| Ingonish River \| ING \| North America \| Nova Scotia \| 46.62 \| -60.45 \| 29 \| \| Inhabitants River \| INH \| North America \| Nova Scotia \| 45.60 \| -61.23 \| 27 \| \| La Have River \| LAH \| North America \| Nova Scotia \| 44.37 \| -64.50 \| 22 \| \| Mabou River \| MAB \| North America \| Nova Scotia \| 46.04 \| -61.31 \| 27 \| \| North Aspy \| NASP \| North America \| Nova Scotia \| 46.83 \| -60.61 \| 29 \| \| Northeast Margaree \| MNE \| North America \| Nova Scotia \| 46.47 \| -60.92 \| 12 \| \| North River(NS) \| NRH \| North America \| Nova Scotia \| 45.38 \| -63.31 \| 22 \| \| River Philip \| RPH \| North America \| Nova Scotia \| 45.59 \| -63.82 \| 17 \| \| Sheet Harbour west \| WES \| North America \| Nova Scotia \| 44.95 \| -62.59 \| 28 \| \| Southwest Margaree \| MRS \| North America \| Nova Scotia \| 46.24 \| -61.12 \| 14 \| \| Stewiacke River \| STW \| North America \| Nova Scotia \| 45.14 \| -63.38 \| 22 \| \| Morells River \| MOR \| North America \| PEI \| 46.30 \| -62.71 \| 18 \| \| Northeast Complex-1(PEI) \| NEP \| North America \| PEI \| 46.45 \| -62.21 \| 27 \| \| Northeast Complex-2(PEI) \| NET \| North America \| PEI \| 46.38 \| -62.57 \| 24 \| \| Northwest Complex (PEI) \| NWP \| North America \| PEI \| 46.63 \| -64.04 \| 17 \| \| South Central (PEI) \| SCP \| North America \| PEI \| 46.28 \| -63.49 \| 14 \| \| à Mars River \| a_Mars \| North America \| Quebec \| 48.34 \| -70.87 \| 26 \| \| Corneille River \| COR \| North America \| Quebec \| 50.28 \| -62.88 \| 28 \| \| Madeleine River \| MAD \| North America \| Quebec \| 49.23 \| -65.32 \| 28 \| \| Matapedia River \| MAT \| North America \| Quebec \| 48.18 \| -67.14 \| 15 \| \| Natashquan River \| NAT \| North America \| Quebec \| 50.12 \| -61.80 \| 28 \| \| Rivire Aux Rochers \| ARO \| North America \| Quebec \| 50.00 \| -66.86 \| 48 \| \| Rivire de la Trinite \| TRI \| North America \| Quebec \| 49.42 \| -67.30 \| 49 \| \| Saint-Jean (NorthShore) SJQ8 \| SJQ \| North America \| Quebec \| 50.28 \| -64.33 \| 28 \| \| Altaelva \| Alta \| Europe \| Norway \| 69.97 \| 23.37 \| 19 \| \| Ardalselva \| Arda \| Europe \| Norway \| 59.14 \| 6.17 \| 18 \| \| Argardsvassdraget \| Arga \| Europe \| Norway \| 64.31 \| 11.22 \| 13 \| \| Aroyelva \| Aroy \| Europe \| Norway \| 61.27 \| 7.17 \| 16 \| \| Aursunda \| Aurs \| Europe \| Norway \| 64.37 \| 11.39 \| 18 \| \| Beiarvassdraget \| Beia \| Europe \| Norway \| 67.03 \| 14.58 \| 15 \| \| Borselva in Porsanger \| Bors \| Europe \| Norway \| 67.03 \| 14.58 \| 17 \| \| Daleelva Hoyangervassdraget \| Dale \| Europe \| Norway \| 61.22 \| 6.07 \| 12 \| \| Dalselva in Dale \| Dals \| Europe \| Norway \| 61.36 \| 5.40 \| 17 \| \| Eidfjordvassdraget \| Eidf \| Europe \| Norway \| 60.47 \| 7.07 \| 21 \| \| Eira \| Eira \| Europe \| Norway \| 62.68 \| 8.12 \| 18 \| \| Elvegardselva (Bjerkvik) \| Elve \| Europe \| Norway \| 68.55 \| 17.56 \| 16 \| \| Enningdalselva \| Enni \| Europe \| Norway \| 58.98 \| 11.47 \| 15 \| \| Etneelva \| Etne \| Europe \| Norway \| 59.67 \| 5.93 \| 16 \| \| Flekkeelva \| Flek \| Europe \| Norway \| 61.31 \| 5.35 \| 19 \| \| Forsavassdraget \| Fors \| Europe \| Norway \| 68.27 \| 16.63 \| 17 \| \| Gaula in Sor-Trondelag \| GauST \| Europe \| Norway \| 63.34 \| 10.24 \| 24 \| \| Gloppenelva \| Glop \| Europe \| Norway \| 61.77 \| 6.20 \| 25 \| \| Hjalma \| Hjal \| Europe \| Norway \| 61.91 \| 5.85 \| 7 \| \| Homla \| Homl \| Europe \| Norway \| 63.41 \| 10.80 \| 18 \| \| Jolstra \| Jols \| Europe \| Norway \| 61.46 \| 5.83 \| 19 \| \| Komagelva \| Koma \| Europe \| Norway \| 70.24 \| 30.25 \| 17 \| \| Lakselva in Porsanger \| Lakj \| Europe \| Norway \| 70.08 \| 24.92 \| 5 \| \| Laksjohka \| Laks \| Europe \| Norway \| 70.06 \| 27.55 \| 16 \| \| Laukhellevassdraget \| Lauk \| Europe \| Norway \| 69.23 \| 17.86 \| 17 \| \| Malselvvassdraget \| Mals \| Europe \| Norway \| 69.27 \| 18.51 \| 16 \| \| Mana \| Mana \| Europe \| Norway \| 62.54 \| 7.44 \| 14 \| \| Maskejohka \| Mask \| Europe \| Norway \| 70.28 \| 28.15 \| 1 \| \| Namsen \| Nams \| Europe \| Norway \| 64.46 \| 11.52 \| 13 \| \| Nausta \| Naus \| Europe \| Norway \| 61.51 \| 5.72 \| 10 \| \| Numedalslagen \| Nume \| Europe \| Norway \| 59.03 \| 10.06 \| 16 \| \| Reipaga \| Reip \| Europe \| Norway \| 66.91 \| 13.63 \| 17 \| \| Repparfjordelva \| Repp \| Europe \| Norway \| 70.45 \| 24.32 \| 17 \| \| Risfjordvassdraget \| Risf \| Europe \| Norway \| 70.98 \| 28.17 \| 17 \| \| Roksadalsvassdraget \| Roks \| Europe \| Norway \| 69.05 \| 15.87 \| 19 \| \| Ryggelva \| Rygg \| Europe \| Norway \| 61.78 \| 6.13 \| 17 \| \| Saltdalsvassdraget \| Salt \| Europe \| Norway \| 67.10 \| 15.42 \| 7 \| \| Sandfjordelva in Gamvik \| Sand \| Europe \| Norway \| 71.05 \| 28.05 \| 17 \| \| Skienselva \| Skie \| Europe \| Norway \| 59.13 \| 9.63 \| 17 \| \| Skipsfjordvassdraget \| Skip \| Europe \| Norway \| 70.16 \| 19.80 \| 17 \| \| Soya \| Soya \| Europe \| Norway \| 62.89 \| 8.54 \| 17 \| \| Suldalslagen \| Suld \| Europe \| Norway \| 59.48 \| 6.25 \| 16 \| \| Surna \| Surn \| Europe \| Norway \| 62.97 \| 8.67 \| 20 \| \| Sylteelva in Fraena \| Sylt \| Europe \| Norway \| 62.84 \| 7.27 \| 18 \| \| Todalselva (Toaa) \| Toda \| Europe \| Norway \| 62.82 \| 8.70 \| 17 \| \| Tressa \| Tres \| Europe \| Norway \| 62.52 \| 7.13 \| 18 \| \| Vestre Jakobselve \| VeJa \| Europe \| Norway \| 70.11 \| 29.33 \| 22 \| \| Vigda \| Vigd \| Europe \| Norway \| 63.31 \| 10.18 \| 17 \| \| Vikedalselva \| Vike \| Europe \| Norway \| 59.49 \| 5.90 \| 13 \| \| Vorma \| Vorm \| Europe \| Norway \| 59.27 \| 6.33 \| 18 \| \| Norwegian Aquaculture \| NAQ \| Europe \| Norway \|  \|  \| 189 \| |  |  |  |  |  |
|  |  |  |  |  |  |
|  |  |  |  |  |  |
|  |  |  |  |  |  |
|  |  |  |  |  |  |
|  |  |  |  |  |  |
|  |  |  |  |  |  |
|  |  |  |  |  |  |
|  |  |  |  |  |  |
|  |  |  |  |  |  |
|  |  |  |  |  |  |
|  |  |  |  |  |  |
|  |  |  |  |  |  |
|  |  |  |  |  |  |
|  |  |  |  |  |  |
|  |  |  |  |  |  |
|  |  |  |  |  |  |
|  |  |  |  |  |  |
|  |  |  |  |  |  |
|  |  |  |  |  |  |
|  |  |  |  |  |  |
|  |  |  |  |  |  |
|  |  |  |  |  |  |
|  |  |  |  |  |  |
|  |  |  |  |  |  |


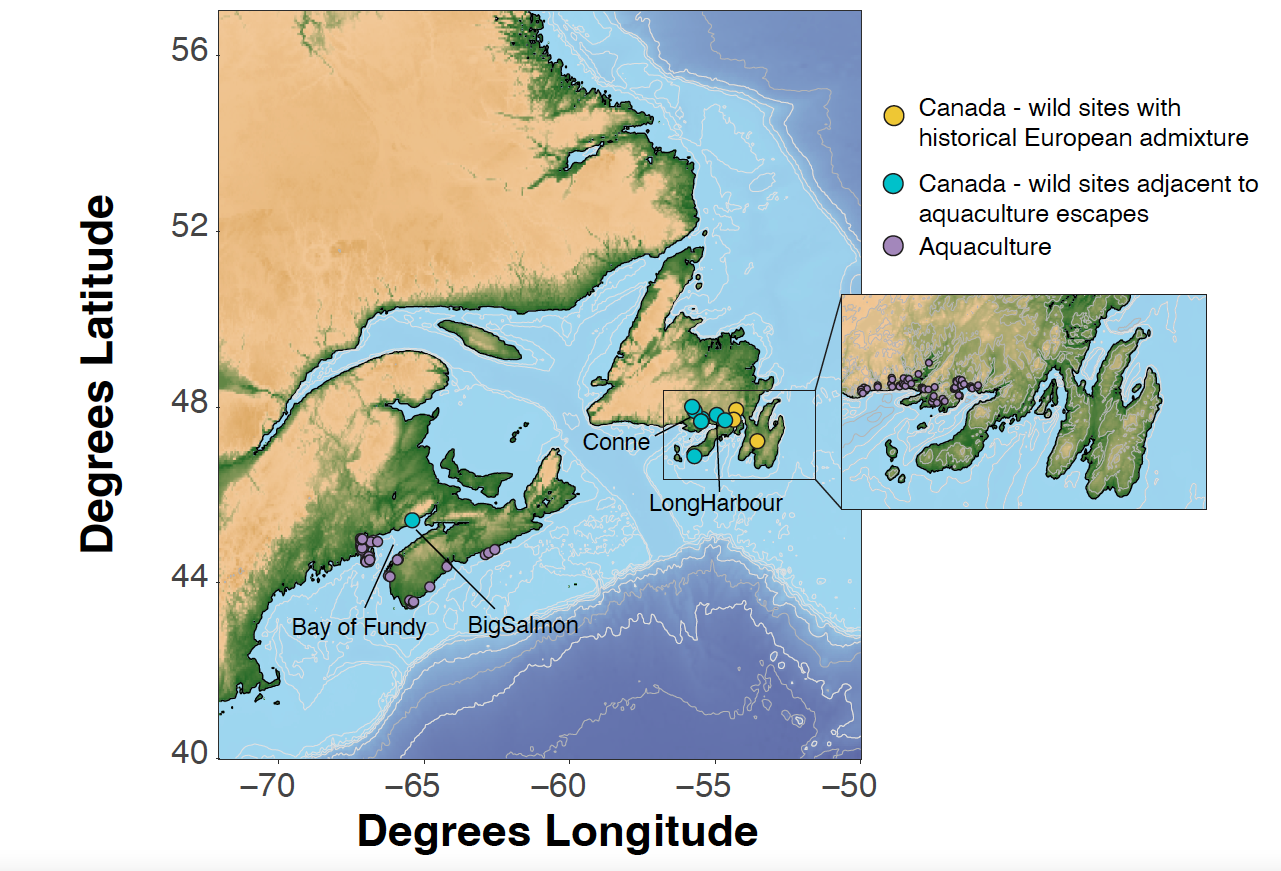


Figure S1. Map of locations where juvenile salmon with elevated European ancestry were detected including both locations near aquaculture cages (blue) and those distant from cages but with evidence for naturally occurring post-glacial secondary contact between North American and European salmon (yellow) as well as aquaculture site locations (purple).


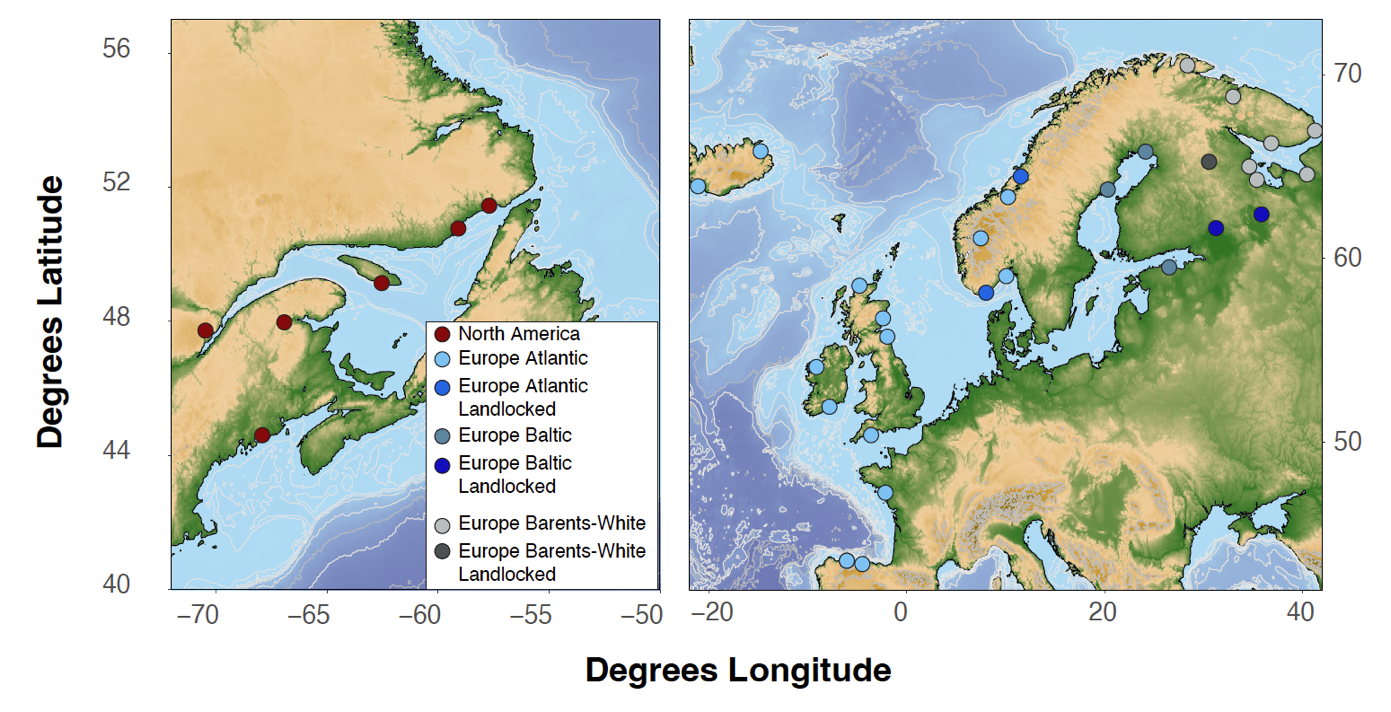


Figure S2. Map of European baseline locations from Bourret et al. (2013) included for comparison (see Figures S3, and S4). Colors indicate main regional genetic groups identified by Bourret et al. (2013).


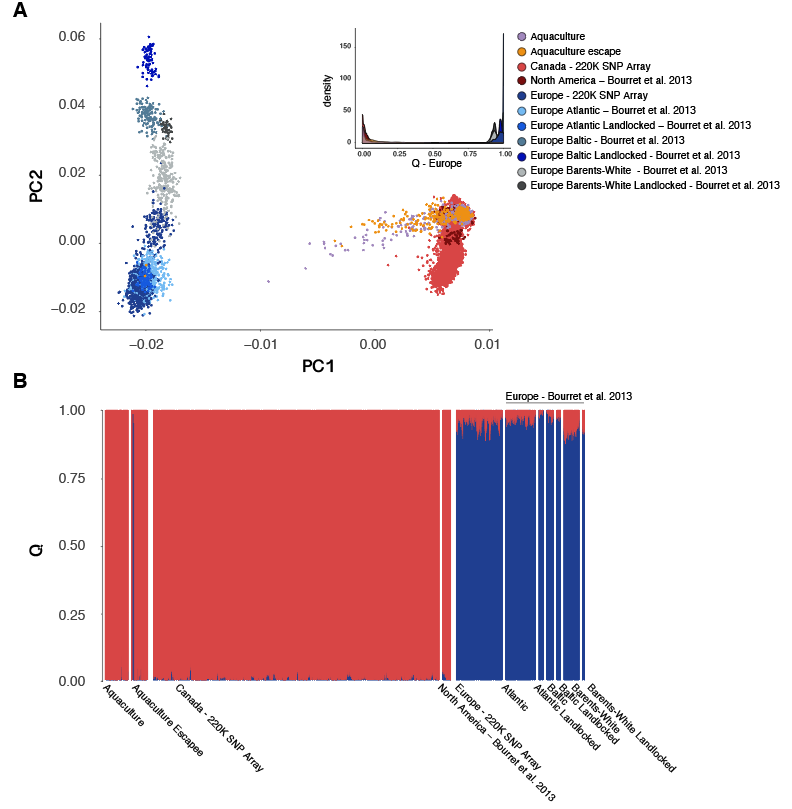


Figure S3. Estimates of European ancestry for North American aquaculture, escapee, and wild collected Atlantic salmon samples using baseline North American and European Atlantic salmon data from Table S1 as well as European and North American SNP data from Bourret et al. 2013 with distinct European groupings indicated (Figure S2) using 1910 SNPs. (A) Principal Component Analysis for aquaculture salmon (purple), escapees (orange), and wild collected Atlantic salmon (red) from this work in comparison to North American and European baselines, inset shows frequency distribution of European q-values colored by group (see below); (B) European admixture (i.e. Q-values) for aquaculture salmon, escapees, and wild collected Atlantic salmon in comparison to North American (red) and European (blue) baselines. See methods for details regarding the baseline samples used for comparison.


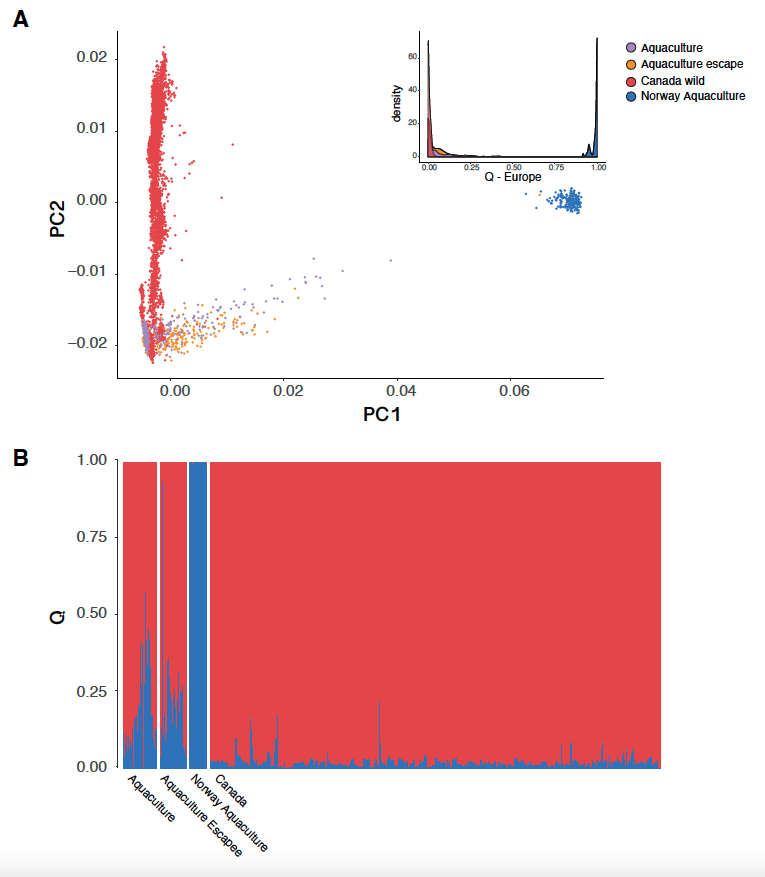


Figure S4. Estimates of European ancestry for North American aquaculture, escapee, and wild collected Atlantic salmon samples using baseline North American (Table S1) and Norwegian aquaculture salmon. (A) Principal Component Analysis for aquaculture salmon (purple), escapees (orange), and wild collected Atlantic salmon (red) from this work in comparison to Norwegian aquaculture salmon, inset shows frequency distribution of European q-values colored by group (see below); (B) European admixture (i.e. Q-values) for aquaculture salmon, escapees, and wild collected Atlantic salmon in comparison to North American (red) and Norwegian aquaculture salmon (blue) baselines.
